# Live and Learn

**Published:** 1852-10

**Authors:** 


*Live and Learn.*—This common expression caught our observation a few days since as the heading of an article, as follows: "Sir J. Percy says, it is quite a mistaken idea to suppose that sugar injures the teeth. No persons have whiter teeth than the negroes, particularly during crop time, and it is equally absurd to suppose that the use of sugar produces worms in children. Worms arise from an insufficiency of salt and bitters in the food of infants; provided these tonics be given, the more sugar is given to a child the greater its health and strength." What a sweet doctrine this is to be sure, to be so utterly incorrect, and great is the pity that such stuff is authenticated to the public as a part to learn. Now we would advise this gentleman to adopt the motto of "*Live and let Live,*" and commend it to all parents who have seen the above article, for they would most certainly destroy the health and happiness of their children by such a course. Sugar is a harmless and beneficial constituent of food in a proper quantity, but in excess produces the most objectionable consequences to many, and some are so peculiarly constituted as to render the use of sugar in almost any form incompatible with good health, particularly dyspeptics giving rise to great flatulency and acidity of stomach, and Dr. Prout affirms that the too free use or abuse of sugar occasions the oxalic acid form of dyspepsia, and often the oxalate of lime calculus, as the common sugar contains a large proportion of lime. Leibeg asserts that sugar is merely, and only, an element of respiration, and not even nutritious, but this we deem incorrect as it is found in almost all kinds of food and would not be placed their without its nutrition was essential, and so we believe it to be, but we would not allow so great an error to go unrestricted, and are not willing to admit that because a few insects and negroes with an occasional white man, have been known to feed largely upon it and continue in health, that it thereby authorizes a general indulgence in an article that requires restriction in its use. That it does direct injury to the teeth is most likely, as it contains in its coarse state, lactic and tannic acids, and again forms combination with the fluids of the mouth which, when held in contact with the teeth, will certainly produce decay. Its indirect injury is not less certain; of course we speak of excess in its use, as whatever will produce a morbid change in the general system will also assuredly affect the mouth and its appendages. That Henry, Duke of Beaufort, should have eaten a pound per day for forty years, is no evidence of its being a wholesome article in larger quantities, but only a proof of an exception to a more well authenticated rule, viz. "that sugar in moderate quantities is a wholesome constituent of food."

